# Alcohol Use Disorder and Chronic Pain – Our experience

**DOI:** 10.1192/j.eurpsy.2025.1895

**Published:** 2025-08-26

**Authors:** S. Momirović, L. Ilić, J. Đokić, I. Nikolić

**Affiliations:** 1 Special Psychiatric hospital “Dr Slavoljub Bakalović”, Vršac; 2 Clinic for Neurosurgery, University Clnical Center of Serbia; 3 Medical Faculty, University of Belgrade, Belgrade, Serbia

## Abstract

**Introduction:**

Chronic painful conditions in alcohol use disorder (AUD) are often seen and represent a significant problem in their therapy. Its incidence is higher than in the general population and is often associated with AUD relapse. Often, they are very manipulative.

**Objectives:**

The aim of our study was the intersection of the state of therapy and therapeutic response in patients with chronic pain and AUD.

**Methods:**

This cross-sectional study includes 25 patients treated at the Department for male alcoholism in the SPH “Slavoljub Bakalovic” in Vršac during their hospitalization. The covered period was from April 1^st^ to August 31^st^ 2024.

**Results:**

During our research, 48 male patients with AUD were treated at our department, and 25 (52.01%) had horonic pain. The average age of the patients was 52 years (24-80), and the duration of symptoms was from 7 months to 20 years (average 8 years and 4 months). Localization was mainly in the area of the lower back (10), lumboischialgia (9) and only in the extremities (6). According to the type of pain, the majority (23) had predominantly neuropathic pain. The average value of pain intensity on the VAS scale was 4 (4.2). All patients were treated with non-steroidal analgesics and benzodiazepines. Along with the mentioned therapy, 9 (36%) patients received a coanalgetic from the group of anticonvulsants and 12 (48%) from the group of antidepressants. Few patients (7) used before and during the hospitalizations supplement based on Mg and vitamin B complex. A good therapeutic response was achieved in 17 patients (reduction of pain on the VAS scale by 2 or more points), partial in 6 patients (reduction of pain on the VAS scale by 1 point). In 2 patients, the prescribed therapy did not reduce pain.

**Conclusions:**

Chronic pain syndromes in AUD is more frequent than in general population, and early detection and good therapy protocol is very important to reduce symptoms. Pain treatment protocol for AUD patients must be made individually for each patient in order to achieve an adequate therapeutic response and avoid interaction with other drugs in the therapy of AUD. With a well-balanced therapy, a good therapeutic response in pain reduction can be achieved.

**Disclosure of Interest:**

None Declared

